# Role of Intestinal Bitter Sensing in Enteroendocrine Hormone Secretion and Metabolic Control

**DOI:** 10.3389/fendo.2018.00576

**Published:** 2018-09-27

**Authors:** Cong Xie, Xuyi Wang, Richard L. Young, Michael Horowitz, Christopher K. Rayner, Tongzhi Wu

**Affiliations:** ^1^Centre of Research Excellence in Translating Nutritional Science to Good Health, The University of Adelaide, Adelaide, SA, Australia; ^2^Institute of Diabetes, School of Medicine, Southeast University, Nanjing, China; ^3^Nutrition and Metabolism, South Australian Health and Medical Research Institute, Adelaide, SA, Australia

**Keywords:** bitter taste receptors, gut hormones, enteroendocrine cells, energy intake, blood glucose, obesity, type 2 diabetes

## Abstract

The gastrointestinal tract stores ingested nutrients in the stomach which are then delivered to the small intestine at a controlled rate to optimize their digestion and absorption. The interaction of nutrients with the small and large intestine generates feedback that slows gastric emptying, induces satiation, and reduces postprandial glycemic excursions. The mechanisms underlying these nutrient-gut interactions are complex; it has only recently been appreciated that the gut has the capacity to detect intraluminal contents in much the same way as the tongue, via activation of specific G-protein-coupled receptors, and that ensuing signaling mechanisms modulate the release of an array of gut hormones that influence gastrointestinal motility, appetite and glycemia. Interestingly, evidence from preclinical models supports a functional link between intestinal bitter taste receptor (BTRs) and gastrointestinal hormone secretion, and the outcomes of recent studies indicate that stimulation of intestinal BTRs may be used to modulate gastrointestinal function, to diminish energy intake and limit postprandial blood glucose excursions in humans. This review summarizes current evidence about the expression and function of intestinal BTRs in relation to enteroendocrine hormone release and discusses the clinical implications of this pathway for the management of obesity and type 2 diabetes.

## Introduction

**Figure 1:**
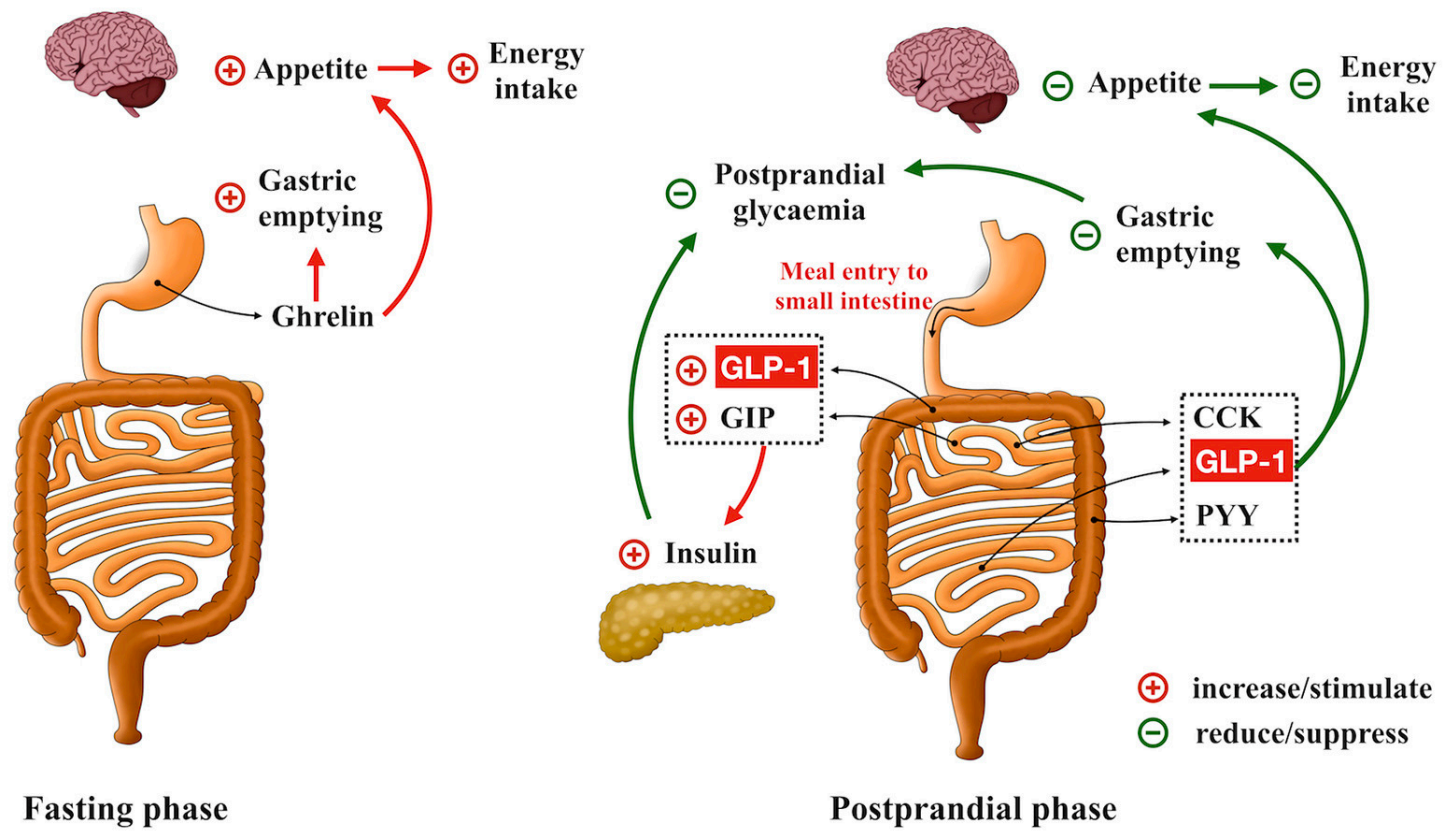
Role of gastrointestinal hormones in the regulation of gastric emptying, postprandial glycemia and energy intake. Ghrelin is secreted during fasting and acts to accelerate gastric emptying, promote appetite and drive energy intake. GLP-1, GIP, CCK, and PYY are released in the postprandial phase. GLP-1 and GIP are the ‘incretin’ hormones, stimulating insulin secretion in a glucose-dependent manner. GLP-1, CCK, and PYY also form intestinal feedback to slow gastric emptying and suppress energy intake.

Recent decades have witnessed the conceptual evolution of the gastrointestinal tract from being solely a site of nutrient digestion and absorption to its recognition as the largest endocrine system in the body - more than 30 peptides are now known to be released from enteroendocrine cells within the gastrointestinal mucosa. These gut-derived hormones communicate with tissues both within and outside the gut, and play a pivotal role in the regulation of metabolic homeostasis. Of particular importance are ghrelin, released from the enteroendocrine Gr-cells (within the stomach); cholecystokinin (CCK), from I-cells (mainly in the upper small intestine); glucose-dependent insulinotropic polypeptide (GIP), from K-cells (largely in the upper small intestine); and glucagon-like pepetide-1 (GLP-1) and peptide YY (PYY), from L-cells (predominantly in the distal small and large intestine) (Figure 1). Ghrelin is secreted predominantly during fasting and is suppressed after meals. It is regarded as a “hunger” hormone that drives food intake and accelerates gastric emptying (1, 2). In contrast, CCK, GIP, GLP-1, and PYY are predominately released postprandially and, in concert, mediate intestinal feedback to limit postprandial glycemic excursions and suppress energy intake (2, 3). In health, GIP and GLP-1 are responsible for the substantially greater insulin response to oral, or enteral, glucose administration when compared with “isoglycaemic” intravenous glucose infusion–the so-called “incretin” effect (4). In type 2 diabetes, the insulinotropic effect of GLP-1 remains relatively intact, although that of GIP is markedly diminished, which may account for the diminished incretin effect in this group (5). GLP-1 also exerts a glucose-dependent glucagonostatic effect (5) and, together with CCK and PYY, acts to slow gastric emptying and suppress energy intake (2). Accordingly, modulation of gut hormone secretion has been actively pursued as a therapeutic option in the management of obesity and type 2 diabetes (5–12). To this end, it has been suggested that a wide array of chemo-sensors expressed on different enteroendocrine cells is responsible for the detection of carbohydrate [e.g., ATP-sensitive K^+^ channel and sodium glucose co-transporter-1 (13, 14)], fat [e.g., G-protein-coupled receptors 119 and 120 (15, 16)] and protein [e.g., oligopeptide transporter 1 and calcium sensing receptor (17, 18)] and associated stimulation of gut hormone secretion. Emerging evidence also attests to the functional importance of “taste” signals arising from intraluminal contents in modulating gut hormone release. For example, blockade of intestinal sweet taste receptors (STRs) by lactisole attenuates glucose-induced incretin hormone secretion substantially in healthy humans (19), although stimulation of STRs (by low-calorie sweeteners) alone appears insufficient to stimulate GIP or GLP-1 secretion in humans (20). Unlike STRs, activation of intestinal bitter taste receptors (BTRs), either by pharmacological BTR agonists or physiological bitter compounds, has been shown to modulate gut hormone secretion in various preclinical and clinical experimental settings, leading to reductions in blood glucose and energy intake (21, 22). In this review, we summarize current evidence relating to the expression and function of intestinal BTRs in relation to enteroendocrine hormone release, as well as the clinical implications of this pathway for the management of obesity and type 2 diabetes.

## Intestinal bitter taste receptors

**Figure 2:**
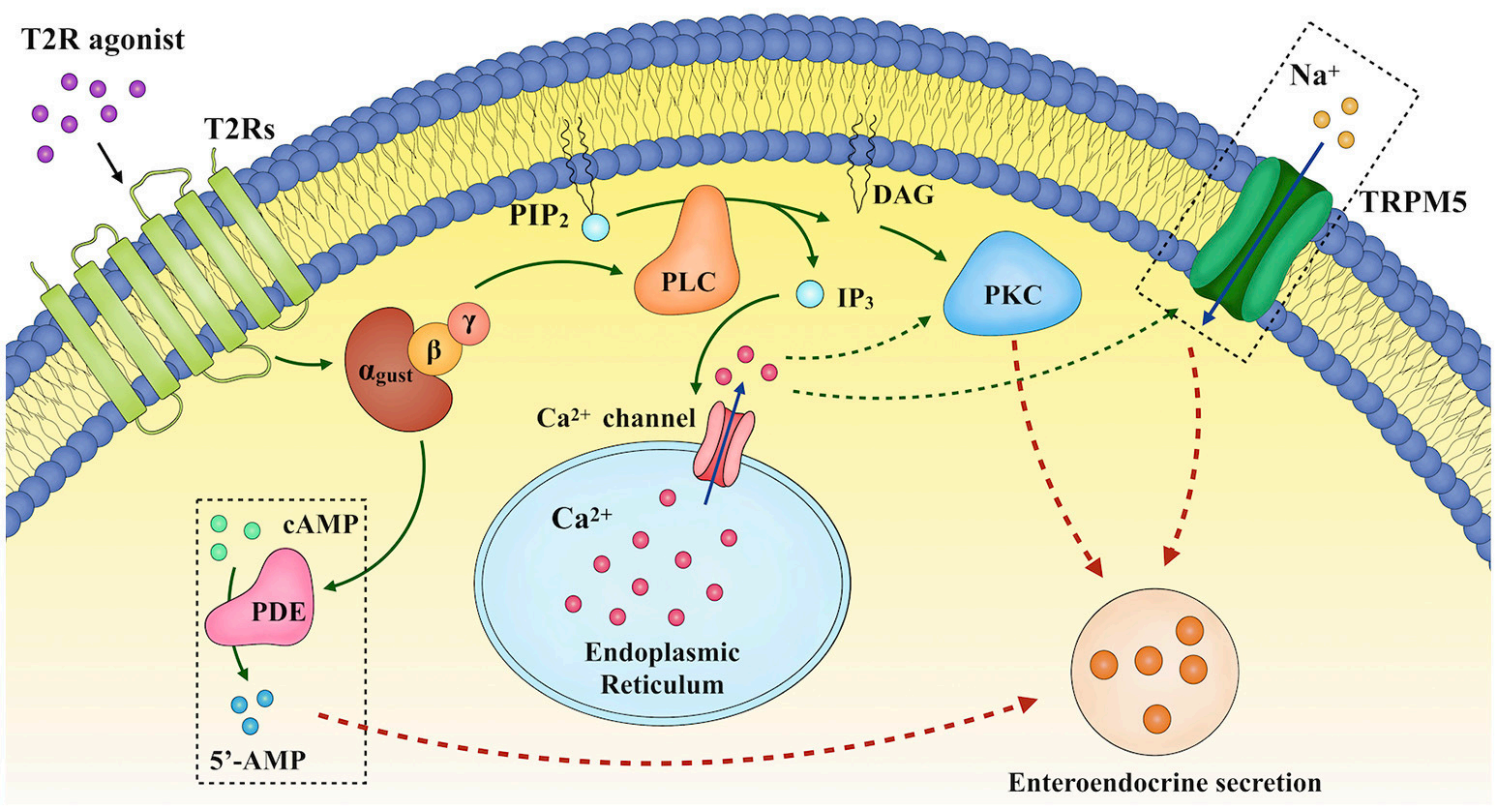
Proposed mechanisms underlying enteroendocrine secretion in response to T2R agonists. Binding of ligands to bitter taste receptors (BTRs) triggers a signaling cascade involving the dissociation of the G-protein gustducin into Gα and Gβγ subunits, activation of phospholipase C β_2_ (PLCβ_2_), production of diacylglycerol (DAG) and inositol 1,4,5-trisphophate (IP_3_), and opening of the transient receptor potential ion channel M5 (TRPM5), thereby leading to the release of intracellular Ca^2+^ ([Ca^2+^]_i_), Na^+^ influx, cellular depolarization and the secretion of neurotransmitters. DAG and [Ca^2+^]_i_ also activate the protein kinase C (PKC) pathway. In addition, increases in intracellular Gα subunit activate phosphodiesterase.

Taste stimuli are detected by a group of specialized G protein-coupled receptors, initially identified in the taste buds of the oral cavity (23). Subtypes of taste 1 receptors heterodimerize to detect sweet (T1R2/T1R3) and umami (T1R1/T1R3) stimuli, while multiple type 2 receptors (T2Rs) are characterized as BTRs and detect bitter stimuli, and may trigger mechanisms which prevent the ingestion and absorption of potentially noxious bitter compounds. Binding of ligands to these taste receptors initiates a signaling cascade involving the dissociation of the G-protein gustducin into Gα and Gβγ subunits, activation of phospholipase C β_2_, production of diacylglycerol and inositol 1,4,5-trisphophate (21, 24, 25), and opening of the transient receptor potential ion channel M5, leading to the release of intracellular Ca^2+^ (21, 24, 26–28), Na^+^ influx (26, 29), cellular depolarization and the secretion of neurotransmitters (28). The increases in intracellular Gα subunit also activate phosphodiesterase to degrade cyclic adenosine monophosphate (cAMP), whereas diacylglycerol and intracellular Ca^2+^ activate the protein kinase C pathway (21, 26) (Figure 2). It has only recently been appreciated that taste receptors and their downstream signaling molecules are also found in extra-oral locations, including the airway, kidney, brain, immune system and the gastrointestinal tract (30, 31). For example, in rodents, inhalation of BTR agonists decreases airway resistance (32), while intravenous administration of the BTR agonist, denatonium benzoate (DB), causes a transient fall in blood pressure (33). The focus of this review, however, is the biology of intestinal BTRs, and in particular their relevance to the secretion of gastrointestinal hormones from enteroendocrine cells.

In a seminal study reported in 2002, Wu et al. demonstrated gene expression of several T2Rs in both the stomach and duodenum of mice and rats using reverse transcriptase-PCR (34). In addition, T2Rs were also found to be expressed on the secretin tumor cell line (STC-1), an enteroendocrine cell model derived from murine enteroendocrine tumors (34). That the exposure of STC-1 to different bitter compounds resulted in a rapid increase in intracellular Ca^2+^ indicated that a functional BTR-sensing system may be present on the enteroendocrine cells (34). These observations were further validated in subsequent studies employing reverse transcriptase- and quantitative-PCR assays on small and large intestinal tissues and enteroendocrine cells of both rodents and humans (Table 1) (25, 42, 43). Consistent with PCR observation, studies using double-labeling immunofluorescence have also shown co-localization of chromogranin A (a cellular marker of enteroendocrine cells) with T2Rs in the mouse small and large intestine (42, 44). More specifically, co-expression of GLP-1 with various T2Rs in human enteroendocrine L cell lines (i.e., HuTu-80 and NCI-h716) and in small and large intestinal tissues has been observed (21, 35, 36, 39). However, the co-expression of T2Rs with enteroendocrine cells containing other hormones is not well characterized in rodents or humans. Moreover, the expression of intestinal BTRs in metabolic disorders has not been consistently reported. In the study reported by Chao et al. (49), the expression of both STR and BTR subtypes were shown to be less in the hypothalamus, brainstem and duodenum in ob/ob mice than C57Bl/6 controls. By contrast, the expression of the BTR, T2R38, in the colonic mucosa was shown to be related directly to BMI in humans, such that the abundance of T2R38 tended to be higher in those who were overweight/obese, when compared to lean subjects (40). In both healthy individuals and patients with type 2 diabetes, the expression of STRs in duodenal biopsy samples did not correlate with BMI or HbA1c, although the dynamic response of STR expression to intraduodenal glucose infusion was found to be impaired in type 2 diabetes (50). Of note, the downstream signaling molecules of taste receptors have also been identified in non-endocrine cells of the gut. For example, α-gustducin and transient receptor potential ion channel M5 are expressed abundantly in subsets of brush cells in mouse and rat gut (51–53). In murine gastric tissue, α-gustducin-expressing brush cells have been found adjacent to ghrelin-releasing Gr-cells (54, 55). Given that the latter are not in direct contact with the intraluminal contents, i.e., “closed-type,” it is possible that brush cells may act as a sensor for intraluminal contents to regulate ghrelin secretion (56).

**Table 1:** Summary of published reports on the presence of different T2Rs in enteroendocrine cells and gastrointestinal tissues in rodents and humans.

**Species**	**Models**	**T2Rs expressed**	**References**
Human	HuTu-80 cell	T2R4, T2R5, T2R13, T2R14, T2R16, T2R38, T2R39, T2R40, T2R44, T2R46, T2R47, T2R49, T2R50, T2R60	(35–37)
	NCI-H716 cell	T2R1, T2R3, T2R4, T2R5, T2R7, T2R8, T2R9, T2R10, T2R13, T2R14, T2R19, T2R20, T2R30, T2R38, T2R39, T2R40, T2R41, T2R45, T2R46, T2R50, T2R60	(21, 24, 38, 39)
	Small intestine	T2R5 T2R14 T2R38	(36, 37, 39)
	Large intestine	T2R1, T2R3, T2R4, T2R5, T2R10, T2R13, T2R38, T2R39, T2R40, T2R42, T2R43, T2R44, T2R45, T2R46, T2R47, T2R49, T2R50, T2R60	(35, 36, 38, 40, 41)
Mouse	STC-1 cells	mT2R102, mT2R104, mT2R105, mT2R106, mT2R107, mT2R108, mT2R109, mT2R110, mT2R113, mT2R114, mT2R116, mT2R117, mT2R118, mT2R119, mT2R121, mT2R122, mT2R123, mT2R124, mT2R125, mT2R126, mT2R129, mT2R130, mT2R131, mT2R134, mT2R135, mT2R136, mT2R137, mT2R138, mT2R139, mT2R140, mT2R143, mT2R144	(25, 42, 43)
	Small intestine	mT2R102, mT2R104, mT2R105, mT2R106, mT2R107, mT2R108, mT2R110, mT2R113, mT2R114, mT2R116, mT2R117, mT2R119, mT2R121, mT2R122, mT2R123, mT2R124, mT2R126, mT2R129, mT2R130, mT2R134, mT2R135, mT2R136, mT2R137, mT2R138, mT2R139, mT2R140, mT2R143, mT2R144	(26, 44–47)
	Large intestine	mT2R108, mT2R113, mT2R117, mT2R118, mT2R119, mT2R125, mT2R126, mT2R131, mT2R135, mT2R136 mT2R137, mT2R138, mT2R140, mT2R143	(26, 46–48)
Rat	Small intestine	rT2R1, rT2R2, rT2R3, rT2R4, rT2R5, rT2R6, rT2R7, rT2R8, rT2R9, rT2R10, rT2R12, rT2R16, rT2R34, rT2R38	(34, 48)
	Large intestine	rT2R, rT2R16, rT2R26	(41)

## Effects of BTR signaling on gut hormone secretion

An increasing number of studies in both preclinical and clinical models have evaluated the effects of BTR agonists on ghrelin, CCK, GLP-1, and PYY secretion, although the specificity of bitter compounds for different T2Rs is poorly defined and the function of intestinal BTR sensing in either obesity or type 2 diabetes has not been thoroughly investigated. In contrast, information regarding GIP secretion in response to BTR stimulation is limited (Table 2).

**Table 2:** Effects of bitter tastants on gut hormone secretion in preclinical and clinical models.

**Hormone**	**Preclinical/Clinical**	***Vitro/Vivo***	**Model**	**Bitter tastants**	**References**
Ghrelin	Preclinical	*vivo*	Mice	Mixture of DB, quinine, PTC, D-salicin	(55)
			Human	HCl quinine 10 umol/kg	(57)
	Clinical			HCl quinine 10 umol/kg	(22)
GLP-1	Preclinical	*vitro*	HuTu-80 cells	Phenylthiourea	(36)
			NCI-716 cells	Berberine	(24)
				1,10-phenanthroline	(39)
				Gentiana scabra	(58)
				DB	(21)
			STC-1 cells	extract from wild bitter gourd	(59)
				Berberine	(25)
		*vivo*	Mice	Extract from wild bitter gourd	(59)
				DB	(21)
				Qing-Hua Granule	(29)
				Gentiana scabra	(58)
	Clinical		Healthy volunteer	Gentiana lutea root	(60)
CCK	Preclinical	*vitro*	STC-1 cells	DB and PTC	(43)
			HuTu-80 cells	H.g.−12 (extract of the plant Hoodia gordonii)	(37)
			Caco-2 cells	PTC	(61)
		*vivo*	mice	Mixture of DB, quinine, PTC, D-salicin	(61)
	Clinical		healthy volunteer	HCl quinine 10 mg	(62)
PYY	Preclinical	*vitro*	NCI-716 cells	DB	(21)

### Ghrelin

The potential role of BTR signaling in the regulation of ghrelin secretion has evaluated in mice and humans, albeit with strikingly different outcomes. In mice, intragastric administration of a mixture of BTR agonists (including DB, phenylthiocarbamide (PTC), quinine and D-[-]salicin) was shown to increase plasma total ghrelin and octanoyl ghrelin levels without affecting ghrelin mRNA expression (55). BTR agonist-induced ghrelin secretion was markedly attenuated in α-gustducin-/- mice. This was consistent with a functional involvement of taste signaling in ghrelin release (55), although α-gustducin is a non-specific downstream signaling molecule and, as discussed, an indirect interaction between brush cells and Gr cells is an alternative possibility. Paradoxically, intragastric gavage of BTR agonists in mice was associated with only a transient increase in food intake during the first 30 min, followed by a sustained suppression of intake over the subsequent 4 h (55). In contrast to the stimulation of ghrelin observed in mice, intragastric administration of another bitter tastant, quinine-hydrochloride (HCl quinine, 10 umol/kg), reduced fasting plasma ghrelin and motilin levels in healthy women (22, 63), associated with increased activity in hedonic and homeostatic brain regions on functional magnetic resonance imaging, and suppressed antral motility and energy intake (22). These observations suggest a role of BTR signaling in communications between the gut and brain in the control of energy intake. However, in another study, intragastric DB at a dose of 1 umol/kg, which suppressed motilin secretion, appetite scores and energy intake, failed to affect either plasma ghrelin or the rate of gastric emptying in healthy women (57). Accordingly, further studies are required to determine the secretory pattern of ghrelin in response to different types and doses of BTR agonists and the associated metabolic effects in humans, including those with obesity and type 2 diabetes.

### CCK

Initial evidence to support the potential for BTR-evoked CCK secretion was reported in STC-1 cells, where both DB and PTC increased intracellular Ca^2+^ and stimulated CCK secretion in a dose-dependent manner (43, 61). Subsequently, steroid glycoside H.g.-12, extracted from the plant *Hoodia gordonii* [which tastes bitter, and has potent appetite-suppressant effects in both animals and humans (64)] was found to induce CCK secretion both *ex vivo* from rat intestine, and from HuTu-80 cells (37). That the effect of H.g.-12 on CCK secretion was abolished by a BTR inhibitor, compound 03A3, supports a functional role of BTR signaling in H.g.-12-induced CCK release (37). While co-expression of BTRs with CCK-secreting I-cells has not been assessed in humans, oral administration of encapsulated HCl quinine (18 mg) was recently reported to increase plasma CCK concentrations and reduce energy intake at an *ad libitum* meal in healthy young individuals (62). Moreover, in this study the magnitude of suppression of energy intake in response to HCl quinine was related directly to the subjects' sensitivity to the bitter taste of PTC (62). These observations warrant further investigation on the potential of targeting the intestinal BTR signaling pathway to stimulate CCK secretion and reduce energy intake in obesity.

### GLP-1 and PYY

Underpinned by the successful clinical application of GLP-1 receptor agonists and dipeptidyl peptisase-4 inhibitors to the management of type 2 diabetes (5, 11, 12), there has been great interest in the potential for BTR agonists to augment L-cell secretion, and thereby increase concentrations of endogenous GLP-1.

At the cellular level, numerous bitter compounds have been reported to induce GLP-1 secretion from enteroendocrine cells via BTR pathways. For example, in both NCI-716 and STC-1 cells, berberine, a natural bitter plant alkaloid commonly used as an antibiotic, was shown to dose-dependently stimulate GLP-1 secretion via T2R38 (24, 25). Similarly, a specific T2R38 agonist, phenylthiourea, induced GLP-1 secretion from HuTu-80 cells, an effect markedly inhibited by silencing of T2R38 with small interfering RNA (36), In contrast, 1,10-phenanthroline stimulates GLP-1 via T2R5 (39), and DB appears to induce GLP-1 secretion via a broad range of BTRs (including T2R4, T2R43, and T2R46 at least), in NCI-h716 cells (21). Furthermore, blockade of BTRs (e.g., by probenecid), or the downstream pathways relating to BTR signaling, including inositol 1,4,5-trisphophate, phospholipase C β_2_, protein kinase C and/or phosphodiesterase, attenuates GLP-1 secretion induced by bitter tastants (21, 58, 59).

In rodents, exposure of the gut to BTR agonists has also been shown to augment plasma GLP-1 levels (21, 36, 58, 59). In acute settings, an intragastric preload of DB prior to enteral glucose administration increased plasma GLP-1 and insulin concentrations (21), slowed gastric emptying (26, 65) and reduced blood glucose (21). Consistent with the role of BTR signaling in GLP-1 secretion, the effect of DB to slow gastric emptying was abolished by co-administration of probenecid (26). Similarly, intragastric administration of PTC has been reported to augment plasma GLP-1 concentrations (36) and slow gastric emptying (26) in mice. The latter effect was, however, not inhibited by probenecid (26). This discrepancy necessitates further investigation to determine whether probenecid sufficiently blocks the BTRs activated by PTC, and whether mechanisms other than BTR-gut hormone pathways account for the slowing of gastric emptying by PTC in mice. In support of the latter, the slowing of gastric emptying induced by a mixture of bitter substances (including PTC) was not affected by concurrent administration of GLP-1 and CCK antagonists in mice (55). In the longer-term (i.e., 4 weeks), intragastric administration of DB remained effective at increasing meal-induced GLP-1 secretion, associated with a reduction in body weight in obese mice, whereas another bitter tastant, quinine, had minimal effect on GLP-1 or ghrelin, despite reducing body weight (66).

While BTRs (e.g., T2R5 and T2R38) have been reported to localize on L-cells in the small and/or large intestine, effects of BTR agonists on GLP-1 secretion are not well characterized in humans. Recently, Mennella et al. evaluated the effect of a single low dose of *Gentiana lutea* root extract encapsulated for release in the small intestine in healthy subjects (60), and observed a tendency for a higher GLP-1 response to a standardized breakfast, and a reduction in post-lunch energy intake compared to placebo (60). Accordingly, additional human studies are needed to evaluate the potential for targeting intestinal BTRs to stimulate GLP-1 secretion.

In contrast to GLP-1, information relating to the effect of BTR agonists on PYY secretion (also released from L-cells) is limited. Although DB stimulates PYY secretion from NCI-H716 cells in a similar manner to GLP-1 (21), this effect has hitherto not been assessed *in vivo*.

## Clinical implications of targeting intestinal BTRs

That BTR signaling is functionally linked to the secretion of hormones integral to the regulation of energy intake and glycemia, as well as the control of gastric emptying, has stimulated substantial interest in targeting this pathway for the management of obesity and type 2 diabetes (publications from clinical studies are summarized in Table 3). The relative absence of calories in bitter compounds represents a substantial asset of this approach.

**Table 3:** Effects of bitter tastants in clinical studies.

**Authors**	**Subjects**	**Bitter tastants and doses**	**Main method**	**Key observation**
(67)	healthy women (*n* = 16)	10 mg quinine sulfate	Sham feeding	Slowed gastric emptying substantially.
(68)	healthy volunteers (*n* = 12)	0.198 mM 500 ml quinine (3.24 mg)	Intragastric administration	Had no effect on gastric emptying.
(62)	healthy volunteers (*n* = 20)	18 mg HCl quinine	encapsulated	Suppressed energy intake; increased CCK secretion; had no effect on gastric emptying.
(60)	healthy volunteers (*n* = 20)	100 mg extracts (from Gentiana lutea root)	encapsulated	Increased GLP-1; suppressed energy intake; had no effect on blood glucose.
(57)	healthy women (*n* = 39)	1 μmol/kg DB	Intragastric administration	Had no effect on gastric emptying; reduced hungry rating and increased satiety ratings.
(63)	healthy women (*n* = 10)	10 μmol/kg HCl quinine	Intragastric administration	Reduced plasma motilin and ghrelin levels; inhibited the antral motility.
(22)	healthy women (*n* = 16)	10 μmol/kg HCl quinine	Intragastric administration	Suppressed energy intake; reduced plasma motilin and ghrelin levels; reduced hungry ratings and increased satiety ratings.

### Effects on energy intake

The impact of BTR sensing in the control of energy intake has been evaluated in both preclinical and clinical studies. Despite variable effects of different BTR agonists on each gastrointestinal hormone, the majority of studies in rodents have reported energy intake to be suppressed following exposure to acute doses of BTR agonists (69–71), although one study reported a transient increase, followed by a sustained suppression of food intake after intragastric administration of a mixture of DB, PTC and salicin (55). Arguably, of greater interest is evidence that intragastric gavage of DB (60 μmol/kg) or quinine (160 μmol/kg) once daily for 4 weeks in high fat-fed obese mice reduced weight gain substantially, and in an α-gustducin-dependent manner (66). In healthy women, a single dose of HCl quinine (10 umol/kg), administrated intragastrically 60 min before an *ad libitum* liquid meal (chocolate milk shake), reduced food intake (346 ± 37 g for HCl quinine vs. 414 ± 46 g for water control), in association with reduced ghrelin levels and increased neural activity in the hypothalamus, hedonic regions, and parts of the medulla associated with appetite homeostasis (22). Consistent with these observations, oral administration of encapsulated HCl quinine (18 mg) also modestly suppressed energy intake at a subsequent *ad libitum* buffet meal (514 ± 248 kcal for HCl quinine vs. 596 ± 286 kcal for placebo) in healthy young subjects (12 females and 8 males) without inducing nausea (62). Likewise, administration of encapsulated bitter compounds derived from *Gentiana lutea* root with a standardized breakfast reduced total daily energy intake by ~20% in healthy individuals (60), while oral insensitivity to the bitter taste of 6-n-propylthrouracil was associated with increased energy intake in female subjects (72). It remains to be determined whether stimulation of intestinal BTRs has the capacity to reduce energy intake and, hence, body weight in obese individuals.

### Effects on blood glucose

The rate of emptying of carbohydrates from the stomach for absorption in the small intestine is a major determinant of the glycemic response to meals (73). In the majority of type 2 diabetic patients with modestly elevated glycated hemoglobin (HbA1c < ~8% or 64 mmol/mol), postprandial glycemia makes the dominant contribution to overall glycemic control (74, 75). In addition, postprandial glycemia is an independent cardiovascular risk factor and predicts all-cause mortality (76), and accordingly, represents a specific target for the treatment of type 2 diabetes. Preclinical models indicate that stimulating intestinal BTRs has the potential to improve blood glucose control. In wild type mice, intragastric administration of DB, PTC or a mixture of bitter compounds slowed gastric emptying substantially (26, 55), while oral administration of DB (1 mg/kg) (21) or *Gentia scabra* root extract (300 mg/kg; containing several bitter compounds such as loganic acid, gentiopicrin and rindoside) (21, 58) in db/db mice was associated with higher GLP-1 and lower blood glucose responses following glucose gavage when compared with saline. In mice fed a high fat diet, oral administration of bitter gourd extract prior to an oral or intraperitoneal glucose load also resulted in higher GLP-1 and insulin levels and lower blood glucose responses (59). That the magnitude of reduction in glycemia was attenuated substantially by concurrent administration of the GLP-1 receptor antagonist, exendin(9–34, 36, 39, 42–44), attests to the importance of GLP-1 to glucose-lowering induced by bitter substances (59).

Hitherto, there is limited information about the effect of BTR agonists on blood glucose in humans. Studies to date have reported inconsistent effects on gastric emptying. In healthy women, sham-feeding with quinine sulfate (10 mg) was reported to slow the emptying of subsequently ingested “electrolyte soup,” when compared to sham-feeding with a “pleasant” strawberry flavoring or control (no sham-feeding) (67). Little et al. compared the rate of gastric emptying of three “test meals” in healthy subjects, consisting of 500 mL water (control) and two bitter-tasting solutions containing either a small dose of quinine (1 mM) or naringin (0.198 mM), delivered via intragastric infusion. Although these doses of quinine and naringin yielded a medium intensity of bitterness during an oral perception test, gastric emptying did not differ between the bitter solutions and water alone (68). More recently, intragastric administration of DB at a dose of 1 umol/kg suppressed appetite sensations, but failed to affect gastric emptying in healthy women (68). However, it remains unclear whether the disparity in findings between studies in mice and humans reflect species differences, or whether the relatively low doses of BTR agonists employed in the human studies were insufficient to interact with L-cells located predominantly in the distal small and large intestine. In the case of GLP-1, infusion of glucose into the duodenum at 2 kcal/min (where glucose is absorbed in the upper gut) elicits minimal GLP-1 secretion, while ileal infusion of glucose at the same rate induces substantial GLP-1 release (77).

The genetic phenotype of GPCRs is now known to be an important determinant of physiological function, may predispose to human diseases (78). There is evidence that polymorphisms of BTR genes that impair the sensitivity to bitterness may be associated with changes in food intake and dysregulation of blood glucose. For example, women with gestational diabetes mellitus exhibited a lower T2R9 gene (rs3741845) frequency, and consumed more meat, dairy and sweet beverages compared to pregnant women without gestational diabetes mellitus (79). Similarly, dysfunction of T2R9 due to a single nucleotide polymorphism is associated with higher blood glucose and insulin responses to an oral glucose tolerance test in Amish individuals with and without type 2 diabetes (38). In German individuals without type 2 diabetes, variations in the T2R38 gene (rs713598, rs1726866 and rs10246939) are also reported to have significant associations with body composition in women, and the glycemic response to oral glucose in men (80).

## Conclusions and prospective views

In recognition of the pleiotropic actions of gastrointestinal hormones in the regulation of metabolic homeostasis, exogenous peptides or mimetics (e.g., GLP-1 receptor agonists and GLP-1/GIP dual receptor agonists) are under rapid development within the pharmaceutical industry to better manage both type 2 diabetes and obesity. This approach, however, is often limited by cost, side effects (predominantly gastrointestinal symptoms), and suboptimal efficacy (particularly for obesity). Dietary strategies to modulate endogenous gastrointestinal hormone secretion represent an alternative that shows substantial promise. For example, consuming a nutrient ‘preload’ prior to the main meal has been shown to reduce postprandial blood glucose in both health and type 2 diabetes by stimulating GLP-1 secretion in advance of the meal, and by slowing gastric emptying (10, 81, 82). However, this approach entails additional energy intake associated with the preload. Modulation of gastrointestinal hormone secretion by low- or non-caloric compounds, such as bitter tastants, would therefore be advantageous compared with nutrient preloads.

There is a large body of preclinical studies that provide compelling evidence of a functional BTR signaling system in enteroendocrine cells, the effects of non-nutritive BTR agonists on enteroendocrine hormone secretion, and the potential for stimulating intestinal BTRs to suppress energy intake and reduce postprandial glycemic excursions (59, 66). However, there are only a handful of clinical studies in healthy subjects (mostly females) that have evaluated the effects of BTR signaling on gut hormone secretion and associated metabolic effects, and no studies in patients with obesity and/or type 2 diabetes. Moreover, the doses of BTR agonists administered in human subjects have been low, probably because bitter tastants are considered to be potentially toxic and aversive (28). Bitter taste perception in the mouth is unpleasant, and naturally serves as an aversive signal for the termination of eating. However, stimulation of intestinal BTRs by administration of different BTR agonists directly into the stomach or duodenum, thereby bypassing oral perception, has not been reported to cause any adverse effects in preclinical models and healthy subjects. Nevertheless, the tolerability of BTR agonists at higher doses remains to be established.

Relative to STRs (T1R2/T1R3) and umami taste receptors (T1R1/T1R3), the biology of BTRs (T2Rs) appears to be more complex due to their diversity. Moreover, expression of BTRs varies substantially along the gastrointestinal tract. For example, T2R2 and T2R6 showed higher expression in gastric than duodenal mucosa in rats (34), whereas in mice, T2R118 and T2R131 are expressed abundantly in the colon, but minimally in the duodenum and jejunum (46). As summarized in Table 1, multiple T2Rs are often co-expressed on the same enteroendocrine cell. However, the relative importance of each has not been characterized. Accordingly, it remains to be determined whether the expression of T2Rs also exhibits regional specificity, in a similar pattern to enteroendocrine cells and, therefore, whether more targeted delivery of BTR agonists is needed for effective stimulation of enteroendocrine hormone secretion. Notably, physiological bitter substances, including bile acids and products of digestion (e.g., amino acids), are abundantly present in the gut after a meal; it is also important, therefore, to understand the physiological role of intestinal bitter taste sensing in the regulation of gastrointestinal hormone secretion, appetite and postprandial glycemia.

## Author contributions

CX, XW, RY, MH, CR and TW were all involved in conception, design and writing of the manuscript. All authors have approved the publication of this final version of the manuscript.

### Conflict of interest statement

The authors declare that the research was conducted in the absence of any commercial or financial relationships that could be construed as a potential conflict of interest.
